# Spontaneous Evisceration of Small Bowel in Incisional Hernia

**DOI:** 10.7759/cureus.22374

**Published:** 2022-02-18

**Authors:** Snehasis Das, Oseen Shaikh, Naveen Kumar Gaur, Gopal Balasubramanian

**Affiliations:** 1 Surgery, Jawaharlal Institute of Postgraduate Medical Education and Research, Puducherry, IND

**Keywords:** meshplasty, spontaneous rupture, laparotomy, evisceration, incisional hernia

## Abstract

Spontaneous rupture of an incisional hernia leading to the evisceration of the intra-abdominal organs is one of the malefic complications seen in these patients. In addition to its rarity, it gets accompanied by possible lethality in the form of incarceration, sequential strangulation, necrosis, and eventual gangrene. If not treated aptly, the clinical scenario could lead to a life-threatening condition with a delay in timely intervention. With less than 20 documented cases, herein, we report a 48-year-old female with a previous history of a midline laparotomy who presented to us with an acute spontaneous evisceration of the small bowel. The patient was immediately decided on surgical management with exploratory laparotomy, adhesiolysis, and primary repair of the abdominal wall defect. Postoperatively, the patient improved without any complications.

## Introduction

An incisional hernia can be defined as an incomplete abdominal wall defect that develops at the site of maximum tension on a previous surgical scar of a full-thickness abdominal wall incision [1]. The highest give-away has been seen to be centered around the infraumbilical domain because of the absence of the posterior rectus sheath as an extra protection layer [1]. Although incisional hernia is fairly prevalent in approximately 20% of laparotomies, spontaneous evisceration due to factorial or non-factorial rupture of the hernia is an infamous rarity [2]. Most of the causalities infer a precipitating factor that would predispose to the occurrence of the phenomenon and, in the absence of congruous treatment, would contribute to the aggravated mortality. We presented a 48-year-old female with spontaneous evisceration of the bowel, managed by exploratory laparotomy, reduction of the eviscerated small bowel, and the primary repair of the defect.

## Case presentation

**Figure 1:**
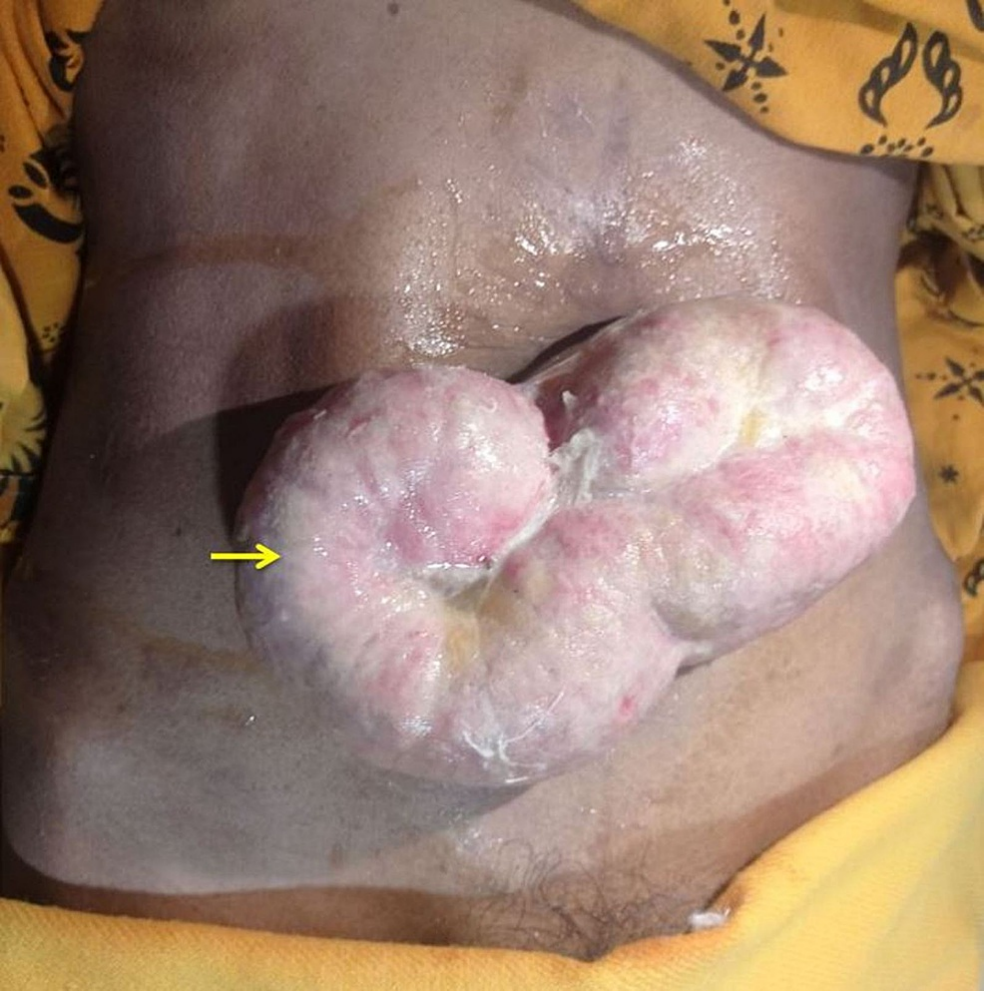
Clinical image showing eviscerated small bowel loop through the incisional hernia site defect.

A 48-year-old female presented with a slow, progressive swelling in the umbilicus for ten days, which spontaneously eroded at the top to expel the bowel in one day. The patient did not have a fever, obstipation, or vomiting. She had a history of midline laparotomy 15 years ago; however, she did not remember the exact reason of the surgery, and there were no records available. On examination, she was poorly built with mild pallor. The patient’s vitals were stable. Examination of other systems was within normal limits. Abdominal examination was suggestive of a 5 cm × 3 cm incisional defect with eviscerated small bowel covered with slough in the infraumbilical region (Figure 1).

Rectal examination showed a rectum loaded with hard stools with no blood staining. Blood investigations showed hemoglobin of 10.4 g/dl with normal liver and renal function tests. An abdominal x-ray was suggestive of mid-dilated small bowel loops proximal to the incarcerated loop at the umbilicus with a maximal diameter of 3 cm.

**Figure 2:**
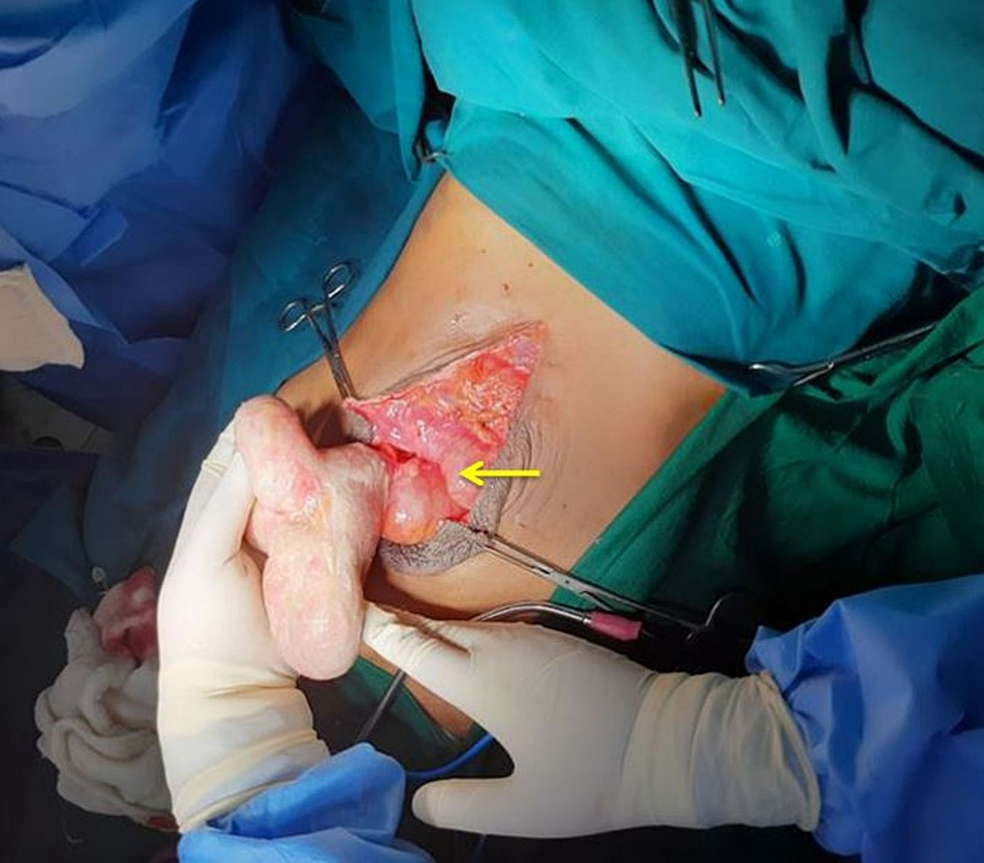
Intraoperative image showing laparotomy incision site (yellow arrow) with an eviscerated small bowel loop.

The patient was admitted as a case of spontaneously ruptured incarcerated incisional hernia. The patient was taken up for an emergency laparotomy to reduce the bowel loop (Figure 2).

The patient was given adequate fluid resuscitation and systemic antibiotics with ceftriaxone and metronidazole. Extensive adhesions were noted between the prolapsed contents and the edge of the defect in the abdominal wall. The herniated loops were released from each other, and the hernia defected. The viability of the contents was confirmed. The herniated loops were identified as viable ileal loops, which were pushed back into the abdomen. The abdomen was closed with tension sutures after removing the redundant skin from the previous incisional hernia site. The patient improved postoperatively without any complications and was discharged after one week.

## Discussion

Incisional herniations have been a part of the surgical abdomen for as long as we know, with spontaneous ruptures being a rare phenomenon. It has been seen to complicate around 20% of laparotomies [2]. It has been reported most commonly in large incisional hernias or recurrent groin hernias where a thinned-out skin vitiates the mechanism for the evisceration [3]. It is usually precipitated by a thin hernia sac and atrophic avascular skin, which causes friction and desquamating dermatitis, leading to ulceration and gaping [4]. The phenomena are propagated by multiple risk factors like bouts of coughing, straining during defecation, heavy weight-lifting, older age, obesity, malignancy and history of wound infection, and repeated wound debridement following initial surgery, which adds on to the causality in the form of a sequential increase in the centrifugal intra-abdominal pressures [5]. Our patient's acute presentation of 10 days makes the above-proposed mechanisms implausible and describes an actual spontaneous burst abdomen.

Complications in the form of adhesions, incarcerations, strangulations, or frank bowel gangrene have been documented well on the side of incisional hernia [6]. Most cases present with intestinal obstruction with completely stable hemodynamics to a collapsed cardiopulmonary system due to vicious shock. Spontaneous rupture has been seen to manifest in bimodality with a gradual and sudden disposition. Sudden ones are caused by a transient exponential increase in the intra-abdominal pressures, which causes the giveaway while in the gradual semblance; it is the prolonged friction ulceration at the dependent part for the sac [7].

The evisceration of the bowel must be treated immediately without any delay as it may cascade into bowel strangulation, gangrene, and perforation [8]. In most cases, the defect acts as a constricting neck, which causes a vicious circle of arterial impedance in the herniated contents. Hence, the contents progress from induced hypoxia from incarceration to gangrenous rupture [8]. In our case, there was the evisceration of the bowel through the incisional hernia site with impending strangulation. However, immediate intervention releasing the constricting ring of the defect prevented the deterioration with the aid of hyperoxygenation and warm mops. Far-out, late misdiagnosed cases carry a malefic prognosis in the form of perforation peritonitis, gut gangrene, and refractory shock, which results in imminent mortality.

If the bowel is viable in emergency surgical ministration, a reduction of the contents into the abdomen followed by a primary meshplasty of the abdominal wall should be performed. Alternatively, primary closure of the abdominal wall with a delayed secondary mesh repair can be performed [9]. In the advent of features of obstruction, it is a case-based decision of the operative surgeons to decide on the viability of a bowel wall with impending features to give a trial of recovery or not. In frank gangrene and bowel necrosis, resection of the involved segment is performed, followed by stoma creation or primary anastomosis depending on the intraoperative vitals, pre-operative albumin levels, bowel wall edema, and amount of intraperitoneal contamination [9]. In cases of increased intra-abdominal pressures, the hernia contents might just be given skin coverage followed by a delayed mesh repair and how the patient's operative site and general condition allow. In our case, the bowel was reduced, and the abdomen was closed without any mesh repair because of the local contamination.

## Conclusions

Spontaneous ruptures of incisional hernias leading to an evisceration of the bowel are rarely reported. Given the associated mortality in the case of delayed treatment due to fulminant metamorphosis, clinicians need to act rapidly and take for surgical repair at the earliest. An early surgical ministration in exploratory laparotomy followed by case-based reduction, stoma or resection, and anastomosis heralds a favorable prognosis. This case is also vital in spreading awareness about this seemingly rare scenario and makes the clinicians aware of the approach to take at the outset to mitigate the accompanying fatalities in the form of life-threatening gut gangrene or perforation peritonitis.
